# RNA-sequencing data analysis of uterus in ovariectomized rats fed with soy protein isolate, 17β-estradiol and casein

**DOI:** 10.1016/j.dib.2016.04.033

**Published:** 2016-04-21

**Authors:** Martin J. Ronis, Horacio Gomez-Acevedo, Michael L. Blackburn, Mario A. Cleves, Rohit Singhal, Thomas M. Badger

**Affiliations:** a Department of Pharmacology & Experimental Therapeutics, LSUHSC-New Orelans, New Orleans, LA, United States; b Arkansas Children’s Nutrition Center, Little Rock, AR, United States; c Department of Biomedical Informatics, UAMS, Little Rock, AR, United States

## Abstract

This data file describes the bioinformatics analysis of uterine RNA-seq data comparing genome wide effects of feeding soy protein isolate compared to casein to ovariectomized female rats age 64 days relative to treatment of casein fed rats with 5 μg/kg/d estradiol and relative to rats treated with estradiol and also fed soy protein isolate. Complete raw data files were deposited in the gene Expression Omnibus (GEO) at NCBI (http:/www.ncbi.nlm.nih.gov.geo/) under the GEO accession number GEO: GSE69819. Data presented here incudes a summary of the differential expression analysis with top 30 genes up- and down-regulated by soy protein isolate (SPI), estradiol (E2) and SPI+E2. Additional functional annotation analysis of KEGG pathways is also presented for each treatment, together with networks of interaction between those pathways. Further interpretation and discussion of this data can be found in the article “Uterine responses to feeding soy protein isolate and treatment with 17β-estradiol differ in ovariectomized female rats” Ronis et al. (2016) [1].

## Specification Table

**Table:** 

Subject area	*Biology*
More specific subject area	*Endocrine Toxicology*
Type of data	*Figure, table, cytoscape networks*
How data was acquired	*Illumina Genome Analyzer IIx*
Data format	*Analyzed*
Experimental factors	*Ovariectomy of female rats to remove endogenous estrogens. Casein vs. Soy Protein Isolate diet, 17*β *estradiol treatment, combined feeding of Soy Protein Isolate.*
Experimental features	*Ovariectomy of female rats at age 50 days. Feeding of purified casein or soy protein isolate diets for 14 days in presence or absence of estradiol treatment at 5 μg/kg/d by s.c. osmotic minipump. RNAseq analysis of uterus mRNA expression patterns.*
Data source location	*Little Rock, AR, U.S.A.*
Data accessibility	Data found in this article and at NCBI (http:/www.ncbi.nlm.nih.gov.geo/) under the GEO accession number GEO: GSE69819

## Value of the data

• First direct in vivo comparison of effects of soy protein isolate and 17β estradiol in the rat uterus at the whole transcriptome level.
• Data will be useful for comparison with other RNA-seq data sets on estrogenic responses in the uterus and other tissues.
• Data will be useful for comparison of estrogen-independent effects of soy feeding on transcriptomic profiles in other tissues.

## 1 Data

Data presented are summaries of the RNA-seq alignment efficiency, differential analysis and tables for the top 30-gene expression changes up or down in the uterus of soy protein isolate fed, estradiol-treated or soy+estradiol treated ovariectomized female rats relative to casein-fed ovariectomized female rats age 64 days after 14 day treatment beginning at age 50 days. Additional functional annotation of KEGG pathways is presented together with enrichment maps for pathways and the functional interaction network from Reactome pathway database.

## 2 Experimental design, materials and methods

### 2.1 Animal care and experimental design

Animal studies received prior approval from the Institutional Animal Care and Use Committee at UAMS. Female Sprague-Dawley rats, postnatal day (PND) 30, were obtained from Charles River Laboratories (Wilmington, MA) and were housed in an AAALAC approved animal facility under a 12 h light-dark cycle. The rats were given *ad libitum* access to semi-purified diets made according to the AIN-93G formula [5] except that corn oil replaced soybean oil and the protein source varied. Half of the animals were fed diets made using casein (CAS) as the protein source, the other half were fed the same diet made with soy protein isolate (SPI) [2], [6]. Rats were fed CAS or SPI diets from PND30 until PND65. On PND50, rats were ovariectomized (OVX) to remove endogenous estrogens. Half of each diet group were subcutaneously infused with 5 μg/kg/d 17β-estradiol (E2) in polyethyleneglycol vehicle and half were infused with vehicle alone via Alzet 2002 mini-osmotic pumps (Alza Corp., Mountain View, CA, USA). Uteri were collected and frozen at −80 °C until analysis. Treatment groups (*N*=6/group), were designed as follows: 1) Control, CAS diet (CAS); 2) CAS-E2, E2 treatment on CAS background; 3) SPI, SPI diet; 4) SPI-E2, E2 treatment on SPI background [1].

### 2.2 Poly-A RNA isolation and preparation of RNA-seq libraries

Total RNA was isolated from each uterus using RNeasy-mini columns (Qiagen, Valencia, CA), including on-column DNAse digestion. RNA quality and integrity was confirmed by A260/A280 ratio (>1.9) and visualization using Experion RNA Std-Sens chips (BioRad, Hercules, CA). Three biologically separate pools containing equal amounts of RNA from 2 individual uteri were utilized. Thus *N*=6 uteri samples were represented over three biological replicate pools. RNA-seq libraries were constructed as follows. Poly-A RNA was isolated from 5 μg of total RNA using Dynabeads® mRNADirect kit (Invitrogen, Carlsbad, CA) and procedures described previously [2]. Briefly, poly-A RNA was captured by addition of 100 μl of Oligo-(dt)25 Dynabeads in 150 μl of lysis buffer (100 mM Tris–HCl, pH 7.5, 500 mM LiCl, 10 mM EDTA, pH 8, 1% LiDS, 5 mM dithiothreitol). The mixture was incubated on a rotary shaker for 20 min at room temperature. mRNA-bead complexes were washed twice with 100 μl of wash buffer A (10 mM Tris–HCl, pH 7.5, 0.15 M LiCl, 1 mM EDTA, 0.1% LiDS), followed by two washes (100 μl each) with wash buffer B (10 mM Tris–HCl, pH 7.5, 0.15 M LiCl, 1 mM EDTA). RNA was eluted from the beads in 11 μl of nuclease free water by heating to 65 °C for 5 min. Following purification, mRNA was sheared to ~150 bp fragments using a Covaris S2 instrument (120 μl volume, 10% DC, 5 intensity, 200 cpb, 7.5 min). Fragmented poly-A RNA was precipitated using sodium acetate/ethanol and reconstituted in 14 μl of nuclease free water. RNA-seq library construction was carried out using NEB-Next reagents (New England Biolabs, Ipswich, MA). First and second strand cDNA synthesis, end-filling using Klenow fragment, and dA-tailing were carried out using manufacturer׳s recommendations. Ligation with Illumina׳s paired-end adapters for multiplexed sequencing was performed with 1 μl of T4 DNA ligase, 0.3 μM of annealed adapters, in a 50 μl reaction volume for 30 min at room temperature. Ligated products were separated using a high-resolution 2% agarose gel (Bio-Rad, Cat # 161-3107), and products around 200 bp (±50 bp) were excised and purified using Qiagen gel extraction kit (Qiagen, Valencia, CA). Size-selected cDNA libraries were amplified using indexed primers containing a 6-bp barcode. PCR was carried out for 12–14 cycles using 29 μl of template, 1 μl of forward and reverse primers (25 μM), and 1 U Phusion high-fidelity DNA polymerase (New England Biolabs, Ipswich, MA). PCR products were purified using Qiaquick PCR purification columns (Qiagen, Valencia, CA) and eluted in 30 μl final volume. A small aliquot (~1 μl) was evaluated using DNA 1000 chip (Experion automated electrophoresis, Bio-Rad, Hercules, CA) to confirm the absence of primer-dimers and other spurious products. Quantification of the RNA-seq libraries was done via quantitative real-time PCR using SYBR green chemistry (Kapa Biosystems, Woburn, MA). Diluted libraries (1:10,000) were quantitated using standards ranging from 0.0002–20 pM.

### 3.3 Sequencing, alignment and data analysis

Libraries from the same group were indexed with unique 6-bp barcodes, combined and clustered on a single-read flow cell. Clustered libraries consisting of 36-bp single read plus an index read were sequenced with a Genome Analyzer IIX using TruSeq v5 reagents (Illumina Inc., San Diego, CA). Image analysis including base calls, was performed by Real-time Analysis software (RTA v2.6, Illumina). Individual.qseq files containing reads and base-quality information per tile of the flow-cell were demultiplexed based on their respective barcodes using scripts in the CASAVA v1.7 suite (Illumina, San Diego, CA). After demultiplexing each lane into biological replicates, three (fastq) files were obtained for each experimental condition. Reads’ quality control was performed with the program FastQc v 0.10. Phred quality scores above 25 were considered acceptable for a given nucleotide. To standardize and remove possible uninformative reads, a quality filter was applied using the tool fastq_quality_filter (FASTX toolkit v 0.0.13.2) with parameter values *p*=0.95 and *q*=25 producing an average of approximately 10 million reads per fastq file (see Table 1).

Reads were aligned to the rn4 rat genome using TopHat v. 2.0.9 and Bowtie 2.1.0 and have an average alignment rate of 79.53 percent. To assemble individual transcripts from RNA-seq reads the program Cufflinks (v 2.1.1) [7] was used. The statistical model for differential expression included in the program Cuffdiff (part of Cufflinks) was used to select genes with significant changes in the summed FPKM values of their transcripts with an FDR-adjusted *p*-Value less than 0.05. Individual differentially expressed gene lists were generated for each of the diet groups keeping casein as control. The data was further annotated and integrated with routines from the library cummeRbund in R (v 3.1.1). Gene lists were further cleaned up imposing a two-fold change cutoff of the estimated gene-expression FPKM values (Fragments Per Kilobase of exon per Million fragments mapped) on a specific experimental group with respect to the estimated FPKM value of the casein (control) group. Lists of the most extreme differentially expressed genes with these conditions implemented were used for subsequent analysis and are presented in a supplementary file (Supplemental data file 1). Data files were also deposited in the Gene Expression Omnibus (GEO) repository at the National Center for Biotechnology Information (http:/www.ncbi.nlm.nih.gov/geo/) under the GEO accession number GEO: GSE69819. Table 2.

**Fig. 1:**
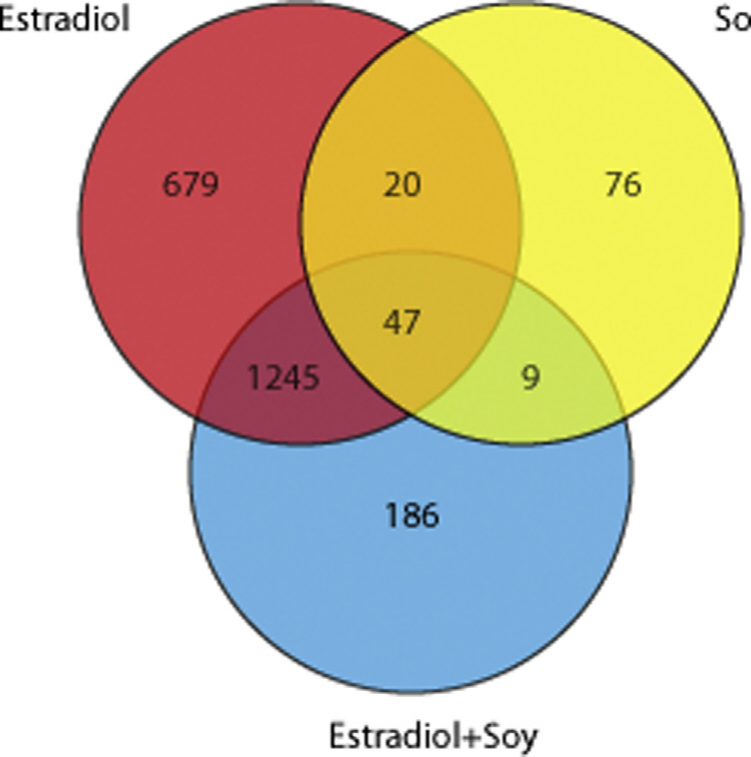
Venn diagram for the transcripts differentially expressed in each experimental group. Cytoscape Figure 1. Soy Enrichment Map. Cytoscape Figure 2. Estradiol Enrichment map. Cytoscape Figure 3. Estradiol+Soy Enrichment map. Cytoscape Figure 4. Soy Functional Annotation Network. Cytoscape Figure 5. Estradiol Functional Annotation Network. Cytoscape Figure 6. Estradiol+Soy Functional Annotation Network.

Functional analysis from our gene lists was performed using the functional annotation tool DAVID (v. 6.7) [3], [4]. The gene id was transformed internally in DAVID, and the rat KEGG pathways for each of the comparisons are presented as a supplement (Supplemental data file 2). We further organized gene lists into a network of interactions using the Enrichment Map plugin in Cytoscape [8]. In those networks, each node represents a gene and edges represent the overlap between pathways. Each network was obtained based on the comparison of the group (soy, estradiol and soy+estradiol) against the control group (casein) with a *p*-Value of 0.05, overlap coefficient of 0.5 without adjusting for false discovery rate (Fig. 1).

Also, we used the Reactome pathway database [9], [10] to build a functional interaction network from the gene lists for each feeding group. The Reactome FI plugin (2013) [11] from cytoscape was used to determine the functional interaction networks.

## Figures and Tables

**Table 1:** Number of reads and mapped reads used for RNA-seq analysis.

Group	Total raw sequences	% Over rep seqs	Phred score (75-perc)	Total cleaned sequences	Total mapped sequences	Overall alignment rate
Caseine 1	12,256,837	0.56	>28	8,648,151	7,444,050	85.70%
Caseine 2	19,588,434	13.19	>32	14,107,321	9,869,491	70.00%
Caseine 3	11,455,598	7.12	>27	8,275,654	6,360,369	76.90%
Caseine+E2−1	12,455,222	0.18	>26	8,620,821	7,273,203	84.40%
Caseine+E2−2	16,073,070	1.38	>28	11,142,669	6,143,389	82.10%
Caseine+E2−3	14,639,165	13.04	>26	10,188,379	6,791,626	66.70%
Soy 1	12,061,880	0.58	>28	8,856,030	7,601,686	85.80%
Soy 2	14,999,942	1.19	>29	11,133,337	9,525,705	85.60%
Soy 3	15,660,248	2.29	>26	11,490,340	9,680,311	84.20%
Soy+E2−1	11,911,178	0.36	>28	8,855,932	7,615,013	85.90%
Soy+E2−2	18,110,799	12.19	>32	13,675,724	9,744,761	71.30%
Soy+E2−3	12,330,268	7.309	>26	9,096,880	6,899,314	75.80%

**Table 2:** Differentially expressed genes by group and direction of regulation.

Group	Upregulated	Downregulated	Total
Soy	133	19	152
Estradiol	1098	893	1991
Soy+Estradiol	908	579	1487
